# Variation in Assessing Renal Allograft Rejection: A National Assessment of Nephrology Practice

**DOI:** 10.1155/2019/5303284

**Published:** 2019-05-13

**Authors:** John Peabody, Paul Billings, Czarlota Valdenor, Zach Demko, Solomon Moshkevich, David Paculdo, Mary Tran

**Affiliations:** ^1^University of California, San Francisco, Department of Epidemiology and Biostatistics, 550 16th St, San Francisco, CA 94158, USA; ^2^University of California, Los Angeles, Fielding School of Public Health, 650 Charles E. Young Dr. South, Los Angeles, CA 90095, USA; ^3^QURE Healthcare, 450 Pacific Ave, Suite 200, San Francisco, CA 94131, USA; ^4^Natera, Inc., 201 Industrial Rd, San Carlos, CA 94070, USA

## Abstract

**Background:**

The clinical utility of early detection and treatment of allograft rejection is well-established. Despite frequent testing called for by standard of care protocols, the five-year kidney allograft survival rate is estimated to be as low as 71%. Herein, we report on posttransplant care provided to kidney allograft recipients by board-certified nephrologists in the United States.

**Methods:**

We measured clinical practice in a representative sample of 175 practicing nephrologists. All providers cared for simulated patients' status after renal transplant ranging from 30-75 years in age and 3-24 months after transplant. Our sample of nephrologists cared for a total of 525 allograft cases. Provider responses to the cases were reviewed by trained clinicians, and care was compared to evidence-based care standards and accepted standard of care protocols.

**Results:**

Among nephrologists, practicing in settings ranging from transplant centers to community practice, we found that the clinical workup of kidney injury in posttransplant patients is highly variable and frequently deviates from evidence-based care. In cases with pathologic evidence of rejection, only 29.1% (102/350) received an appropriate, evidence-based biopsy, whereas, in cases with no pathological evidence of rejection, 41.3% (45/109) received low-value, unnecessary biopsies.

**Conclusion:**

Clinical care in the posttransplant setting is highly variable. Biopsies are often ordered in cases where their results do not alter treatment. Additionally, we found that misdiagnosis was common as were opportunities for earlier biopsy and detection of rejection. This evidence suggests that better diagnostic tools may be helpful to determine which transplant patients should be biopsied and which should not. This study suggests that nephrologists and transplant patients need better tests than creatinine and proteinuria and less invasive approaches than routine biopsies to determine when transplant patients should be investigated for rejection and additional treatment.

## 1. Introduction

The rate of kidney failure, requiring dialysis or kidney transplant, continues to rise in the United States and other parts of the world [1]. Treatment for kidney failure also consumes a large share of healthcare resources, representing over 7% of Medicare's paid claims and $35 billion in Medicare costs annually [2].

Kidney transplantation is considered the treatment of choice for patients with kidney failure because of the increased life expectancy and higher quality of life [3]. Allograft transplantation is also more cost-effective than chronic dialysis, with predicted cost per quality adjusted life year (QALY) for HLA-compatible living donor transplantation estimated to be $39,939 compared to dialysis at $72,476 [4].

The clinical concern of the successful transplant patient is rejection. At five-year posttransplant, kidney allograft survival is as low as 71% [5]. Standards of care protocols recommend regular surveillance for detecting and treating early rejection, which is done by checking creatinine and urine proteinuria and/or by routine biopsy at regular posttransplant intervals [6].

The clinical utility of early detection and treatment of allograft rejection is well-established [7, 8]. We investigated posttransplant practices among nephrologists caring for kidney transplant patients across the U.S. We were particularly interested in scenarios with rejection with only modest or no elevation of their creatinine and patients with elevated creatinine from causes other than rejection.

## 2. Methods

We conducted a prospective, cross-sectional study of the evaluation and care of posttransplant renal allograft patients among a nationally representative sample of nephrologists. We asked board-certified and/or fellowship trained nephrologists to care for three different types of posttransplant cases that reflect a typical post-renal transplant population. We used Clinical Performance and Value (CPV) simulated patients to measure provider practice in three patient types. From their care of the CPV patients, we summarized and compared how nephrologists examined, worked up, diagnosed, and treated posttransplant patients.

*Clinical Performance and Value Vignettes.* We created nine CPV cases, divided into one of three patient case types: (1) active rejection with a moderate creatinine increase and mild to moderate proteinuria, (2) subclinical rejection in a patient with no change in their creatinine, and (3) a patient with an elevated creatinine from another nephrotoxic insult but with no rejection. These nine cases and three subtypes are summarized in Table S1.

CPV simulated patients have been validated against standardized patients and are known to reflect actual care (See Supplement Box 1 for details on CPVs) [9]. CPV patients have been used extensively in a variety of studies over many years to evaluate and compare clinical practice [10, 11]. In a CPV, physicians make inquiries of the patient, review histories, and order laboratory tests and procedures just as they would in an actual patient visit. These open-ended queries in the CPVs are divided into four domains of care: (1) performing a physical, (2) ordering diagnostic workup, (3) making a diagnosis, and (4) determining a treatment plan and follow-up. Each vignette has between 57 and 66 evidence-based criteria evaluated. Scoring is reported as a percentage of the items requested by the participant which align with these criteria. To score these vignettes, two physicians—working independently—compared a physician's case responses against explicit evidence-based, predetermined criteria with a third physician adjudicating in the case of a disagreement on any of the individual criteria. Because all physicians are caring for the same set of patients, CPV vignettes adjust for case-mix variation and provide a clear measurement of clinical practice variation [12].

*Physician Selection.* Between November and December 2018, we randomly recruited the study participants from a list of over 10,000 practicing nephrologists. The recruitment lists were sourced from relevant physician contact files, including workforce databases, list serves, and rosters of medical associations, hospitals, professional organizations, and national conferences. Eligible participants had to (1) be physicians either board-certified or fellowship trained in nephrology, (2) have between two and 40 years of post-residency or post-fellowship practice, and (3) have an active panel of at least 5 renal allograft patients.

Those who met the eligibility criteria and completed a 15-question screener were invited to participate. Enrollment continued until 170 or more physicians were enrolled. We stratified our recruitment so that physician characteristics, including regional geography, age, gender, and practice size, were representative of the nephrologist workforce nationally (see Table S2).

*Analysis.* The primary outcomes were to evaluate how often rejection was correctly diagnosed, under what conditions a biopsy was performed, and whether treatment to reduce rejection was appropriate, i.e., evidence-based. We further sought to determine how assessment practices compared to evidence-based guidance and health care utilization and costs associated with workup and treatment. Chi-squared tests and logistic regression modeling were used for analyses involving binary outcome variables. All analyses were conducted in Stata 14.2.

*Ethics.* This study was conducted in accordance with ethical standards, approved by the Advarra Institutional Review Board, Columbia, MD, and listed in clinicaltrials.gov (NCT03765203). Informed consent was obtained from all participants.

## 3. Results

*Physician-Practice Survey.* From lists of over 10,000 practicing nephrologists, we serially recruited 195 nephrologists who agreed to participate. Among these, 17 did not meet the eligibility criteria and 3 others declined to participate further, leaving a total of 175 who were enrolled into the online study. Prior to doing the cases, each physician was asked to complete a brief physician questionnaire on their background and current practice setting (Table 1). 98.3% were board-certified in nephrology and 1.7% were board-certified in internal medicine only but completed a nephrology fellowship. By age, 20.6% were under 40 years old, 56.0% were between 40 and 54 years, and 23.4% were 55 and older. Like the nephrology workforce in general, the majority (81.7%) of study participants were male. On average, all participants had 14.6±8.1 years of practice experience and currently care for 197 renal posttransplant patients annually. 80.2% work in an urban location. Over 40% of providers worked at four or more practice locations but only 14.9% worked in a hospital-based practice. 60% worked in a transplant center and, of these, 20.0% reported that their center performed routine surveillance biopsies for all of their posttransplant patients and 52.4% performed routine biopsy for only selected patients.

Overall, each of the 175 physician participants cared for three CPV patients for a total of 525 CPV simulated cases completed. Among these cases, we evaluated diagnostic accuracy, appropriate and low-value biopsy rates, how practice compared to protocols, and the costs of care.

*Diagnostic Accuracy*. We found that providers correctly identified rejection (both active and subclinical) 34.0% of the time. There was, however, a significant difference in detection based on whether the rejection was active or subclinical (57.5% vs. 10.3%, p<0.001). In posttransplant patients who had an elevated creatinine due to other causes, the correct diagnosis was reached 79.4% of the time.

*Biopsy Rates.* Among the cases with clinical signs of rejection, 47.4% went on to biopsy. The results were given to those that did the biopsy and this confirmed these were cases of rejection. Among those with no symptoms or signs of rejection, 10.9% of the patients were biopsied, again securing results that confirmed there was evidence of rejection.

For the 175 cases with an elevated creatinine but ultimately no pathologic evidence of rejection, 36.0% were sent for biopsy. In approximately one third (66/175) of these cases, an evidence-based biopsy was appropriate and biopsy was done 27.3% of the time. However, in the two thirds of cases with an elevated creatinine where a biopsy was not indicated (109 cases) biopsy was done in 41.3% of cases. The difference in biopsy rates between the two groups, while disparate, did not prove to be significant (p=0.074). Of note, in the two thirds of cases where biopsy was not indicated, the biopsy results (expectedly) did not increase providers' diagnostic accuracy (80.4% accuracy for those who did not order a biopsy vs. 77.8% for those who did; p=0.700).

We compared nephrologists working in transplant centers using protocols to nephrologists in transplant centers who did not, and we found no difference in the biopsy rates. In centers using protocols that included surveillance biopsies, biopsies were done 34.8% of the time. In centers using protocols that did not always do routine surveillance, biopsies were done 31.5% of the time. In centers that did not use protocols, biopsies were done 27.3% of the time. There was no statistical difference among these three groups (p=0.634). This nonsignificant trend was robust even after breaking out the rejection cases by active (p=0.550) or subclinical (p=0.090) presentation of rejection.

To determine what provider or practice characteristics made biopsy ordering more likely, we performed a multivariate logistic regression, where the dependent variable was appropriate (evidence-based) biopsy (Table 3). We found that older providers (age 55+) were significantly less likely (O.R. 0.47, 95% CI 0.23-0.99) than nephrologists under 40, while those who worked in a hospital (O.R. 2.81, 95% CI 1.47-5.38) were significantly more likely to order a biopsy.

**Figure 1:**
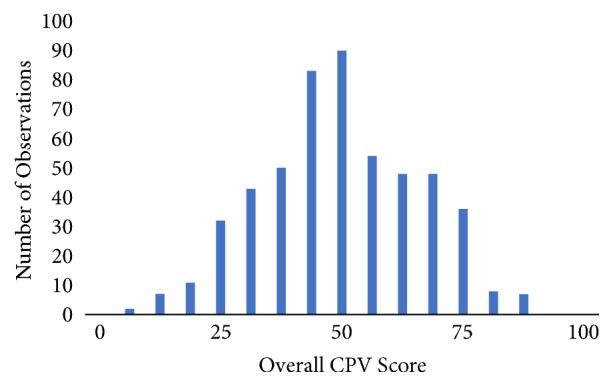
Histogram of CPV scores.

*Practice versus Evidence-Based-Guidance.* Beyond biopsy rates, we found extensive practice variation among the nephrologists as measured against explicit, evidence-based standards (Figure 1). The average overall score for the cases was 46.7%±16.0% and the interquartile range (IQR) was 36.7%-58.6%. Across care domains, providers achieved the highest average score in physical examination (77.7%±22.4%). The scores decreased in subsequent domains: diagnostic workup was 47.8%±33.3% and diagnoses plus treatment scores were 34.2%±24.8% (Table 2). Decreasing scores in the latter domains are unsurprising, as performance relies on obtaining and using the information gleaned from the earlier domains. For example, among cases with rejection, we found that providers ordered IV steroids only 26.3% of the time (41.7% in active cases and 10.9% in subclinical cases, p<0.001). Interestingly, in cases where it was necessary for providers to adjust the tacrolimus dosage, providers appropriately decreased the dose 78.9% of the time but only appropriately increased the dose only 21.9% of the time.

*Cost of Care.* Last, we looked at the utilization and the cost of low-value care. We observed that providers ordered about one (0.9±1.4) low-value diagnostic test, i.e., a test not necessary to reach the correct diagnosis, at a cost of $229±$567 per case. This amounts to $120,000 in potential savings from the 525 cases cared for by the 175 providers in the study for one episode of care. As noted above, 41.3% of nonrejection cases had a biopsy ordered. Using a conservative estimate of $1,482 per biopsy (based on 2018 Medicare prices), this amounts to $67,000 and accounts for over one-half of the $120,000.

## 4. Discussion

In recent years, there has been a decrease in overall rejection rates but this has not led to a significant improvement in long-term graft survival [13]. Timely recognition and treatment of clinically apparent and subclinical rejection remains the foundation for preventing long-term graft loss [14]. We investigated the diagnostic and therapeutic accuracy of practicing nephrologists across the country caring for kidney rejection in posttransplant patients, with particular interest in biopsy rates, evidence-based practice, and the costs of care.

Across three common case types, we found care practices varied widely and often deviated away from evidence-based practice. In clinically active rejection cases with a rising creatinine, only 47.4% providers ordered an appropriate biopsy, 57.7% made the correct diagnosis of active rejection, and 41.7% prescribed the primary guideline-based treatment. In subclinical rejection patients, where there is no clear evidence to biopsy or not, 10.9% of the cases had a biopsy ordered, 10.3% were given the correct diagnosis, and 10.9% received appropriate treatment.

One takeaway from this is that current monitoring tests for assessing renal allograft function (creatinine, urine protein quantification) are insufficient for detecting active rejection early and wholly inadequate for detecting subclinical rejection. In patients with acute kidney injury not from rejection, another key finding is that biopsies were ordered unnecessarily in 41.3% of the cases; these biopsies did not improve diagnostic accuracy though they did increase costs.

Standard practice identifies rejection by either elevated or rising serum creatinine leading to for-cause biopsy confirmation or by routine surveillance percutaneous biopsy. There are serious limitations of either approach for detecting rejection: creatinine is neither specific nor sensitive and biopsy is invasive, expensive, and risky. Neither satisfies the patient's need for early detection at minimal risk or addresses the problem of misdiagnosis, identified in this and other studies [8]. Ideally, fewer biopsies should be ordered in patients where there is no rejection and, for patients with azotemia, more timely and personalized use of immunosuppression therapy. The evidence is mixed whether there is any benefit to treating subclinical rejection [15, 16], although in our study providers who had biopsy-confirmed rejection were more likely to increase immunosuppression.

Timely treatment of subclinical rejection has the potential to improve the long-term outcomes of renal transplant patients. Although the data are mixed, the development of antibody-mediated rejection (ABMR) is a known risk factor for rejection after kidney transplantation. In a recent study, a subclinical variety of ABMR progressed to chronic ABMR [17]. In parallel, others have recommended early treatment of ABMR to improve outcomes [18].

There are a number of limitations to this study. While efforts were made to match demographics of practicing nephrologists in the US, we had a higher representation of men and middle-aged physicians in our final participant population, and we had a majority representation of nephrologists that worked in transplant centers (59.5%) compared to the community. Also, having adjusted for possible case-mix variability, it is possible that there are other clinical scenarios that would have yielded different results. In designing these cases, however, we made a concerted effort to present typical cases representative of a large portion of posttransplant patients. Finally, while the nature of this study does not allow collection of patient outcome data, we have physician practice data using a tool known to reflect actual practice.

At present, no clear guidelines exist that stratify patients into different risk-groups. In general, patients with a higher risk for rejection are simply monitored more closely, and their management is handled solely at the physician's discretion. This leads to highly variable practices that may lead to suboptimal outcomes. A more accurate assay, which helps physicians manage renal allograft health, would be ideal. The ideal test should have clear thresholds indicating rejection versus nonrejection and be able to distinguish rejection from other causes of nephrotoxicity, such as BK viremia and drug toxicity including immunosuppressive agents used to combat rejection [19]. A better test would make it possible, too, to tailor or reduce immunosuppressive and prophylactic antibacterial regimens and improve risk stratification protocols.

## Figures and Tables

**Table 1:** Baseline provider characteristics.

*N*	175

*Male*	81.7%
*Age*	
<40	20.6%
40-55	56.0%
>55	23.4%
*Board certification*	
Internal medicine	1.7%
Nephrology	30.3%
Both	68.0%

*Years in practice*	14.6±8.1

*Region*	
Midwest	17.7%
Northeast	27.4%
South	28.6%
West	26.3%
*Locale*	
Urban	80.2%
Suburban	15.1%
Rural	4.7%
*Employed by practice, *%	77.1%
*Multi-specialty practice*	33.7%
*Medical practice setting (can choose more than one)*	
Accountable care organization	5.7%
Solo practice	4.0%
Group practice	53.7%
Hospital-based	14.9%
Integrated delivery system	3.4%
HMO (network/staff model)	0.6%
Other	1.1%
*Number of practice locations*	
1	20.6%
2	21.1%
3	16.6%
4	13.7%
5	6.9%
6+	21.1%

*Work in renal transplant center*	60.0%
*If yes, routine biopsy surveillance protocol for transplant patients*	
Never	27.6%
Selected patients	52.4%
All patients	20.0%

*Receive quality bonus*	24.6%

*Patient panel characteristics (Mean*±*S.D.)*	
Number of active patients with ESRD	269±414
Number of active patients post renal transplant	197±365
*Payer type*	
Medicare	53.7%
Medicaid	17.2%
Commercial	25.4%
Self	2.5%
Other	1.2%

**Table 2:** Summary of CPV results (N=525).

Variable (n)	Results
*CPV Domain*	
*Overall (525)*	46.7±16.0

*Physical (525)*	77.7±22.4
*Workup (525)*	47.8±33.3
*Diagnosis-Treatment (525)*	34.2±24.8

*Low-value tests, # (525)*	0.9±1.4
*Low-value tests, $ (525)*	$229±$567

*Specific Items*	
*Biopsy of renal allograft*	
Active rejection, clinical (173)	47.4%
Active rejection, subclinical (173)	10.9%
Other, non-rejection (173)	36.0%
*Primary diagnosis*	
Active rejection, clinical (173)	57.7%
Active rejection, subclinical (173)	10.3%
Other, non-rejection (173)	79.4%
*Secondary diagnosis*	46.2%

*IV steroids for active rejection (350)*	26.3%
*Continue mycophenolate mofetil dose (520)*	61.5%
*Tacrolimus Dose in CNI Toxicity*	
Continue current dose (297)	42.4%
Decrease dose (109)	78.9%
Increase dose (119)	21.9%
*Follow-up visit (525)*	24.2%
*Continue medications (525)*	53.7%
*Referral to vascular surgeon when appropriate (66)*	72.7%

**Table 3:** Multivariate regression analyses of appropriate biopsy orders.

	Odds Ratio	[95% Conf.	Interval]
*Male*	0.81	0.44	1.50

*Age*			
40-54	0.69	0.38	1.25
>=55	0.47	0.23	0.99

*Internal Medicine*	2.41	0.50	11.74
*Work in south or northeast*	1.20	0.74	1.93
*Urban practice*	0.82	0.47	1.45
*Work in transplant center*	1.29	0.77	2.16
*Hospital setting*	2.81	1.47	5.38
*Subclinical acute rejection case*	0.15	0.09	0.27
*Constant*	0.95	0.38	2.40

## Data Availability

The cases and data summaries used to support the findings of this study are available from the corresponding author upon reasonable request.

## Supplementary Materials

Supplementary Materials Natera Variability Supplement v1 Mar 23 2019 Box S1.docx: this file contains a detailed description of how the CPV cases were developed and the scientific basis behind CPVs as a simulation tool to measure actual clinical practice. Natera Variability Supplement v2 Mar 24 2019 Table S1.docx: this file contains the descriptions of each of the CPV cases developed for the study, so the interested reader may get a sense for the details of each case. Natera Variability Supplement v25 Jan 21 2019 Table S2.docx: this file listed the national demographic distribution of practicing nephrologists in the United States and compared that against the actual demographic distribution of the providers in the study.
