# Supplementary material for: Variation in Assessing Renal Allograft Rejection: A National Assessment of Nephrology Practice
Source: Int J Nephrol. 2019 May 13;2019:5303284. doi: 10.1155/2019/5303284 (PMC6535838; doi:10.1155/2019/5303284)
Supplement: Supplementary Materials — Natera Variability Supplement v1 Mar 23 2019 Box S1.docx: this file contains a detailed description of how the CPV cases were developed and the scientific basis behind CPVs as a simulation tool to measure actual clinical practice. Natera Variability Supplement v2 Mar 24 2019 Table S1.docx: this file contains the descriptions of each of the CPV cases developed for the study, so the interested reader may get a sense for the details of each case. Natera Variability Supplement v25 Jan 21 2019 Table S2.docx: this file listed the national demographic distribution of practicing nephrologists in the United States and compared that against the actual demographic distribution of the providers in the study. [file 5303284.f1.docx]

**Box S1. Clinical Performance and Value vignette details.**

Clinical Performance and Value (CPV®) vignettes are a validated online tool simulating typical patients that reliably measures clinical performance. CPVs are designed to have the look and feel of a typical clinical encounter. CPVs been featured extensively in the peer-reviewed medical literature^1,2^ and have been validated across multiple clinical areas.^3-6^ Comparison with real-world outcomes shows that a 3% to 5% change in CPV scores is correlated with a significant improvement in actual clinical practice.^7^

CPV cases are developed for a particular area (in this case nephrology) by QURE’s Medical Sciences team who research relevant guidelines and pathways, and review the evidence-based literature. Each case consists of 5 domains: (1) taking the patient’s history (e.g., chief symptom, comorbidities, economic status, etc.); (2) performing a physical examination of the patient (e.g., head, chest, extremities, etc.); (3) ordering diagnostic imaging, procedures, and laboratory work; (4) making a diagnosis; and (5) outlining detailed treatment and providing a follow-up plan.

Providers caring for the simulated CPV patient progress through these domains to ascertain the patient’s condition and providing necessary treatment steps.

Caring for a single CPV patient takes about 20 to 30 minutes (again typical of a clinical encounter) and requires providers to make clinical decisions on the medical information they elicit. Since all providers care for the same set of cases, we are able to benchmark provider actions without the need to adjust for case-mix.

Once completed, CPVs were independently, blindly double-scored by QURE’s Medical Sciences Team using pre-defined, evidence-based scoring criteria and blinded to participant identity. Potential scores range from 0% to 100% within each care domain and in aggregate across the CPV. Higher percentage scores reflect a greater alignment with the evidence-based and other specific recommendations (i.e., guidelines, pathways, ordersets, etc.).

1. Peabody J, Luck J, Glassman P, Dresselhaus TR, Lee M. Comparison of vignettes, standardized patients, and chart abstraction: a prospective validation study of 3 methods for measuring quality. JAMA. 2000; 283(13):1715-1722.
2. Peabody JW, Luck J, Glassman P, et al. Measuring the quality of physician practice by using clinical vignettes: a prospective validation study. Ann Intern Med. 2004;141(10):771-80.
3. Kubal T, Letson DG, Chiappori AA, et al. Longitudinal cohort study to determine effectiveness of a novel simulated case and feedback system to improve clinical pathway adherence in breast, lung and GI cancers. BMJ Open. 2016; 6(9):e012312.
4. Bergmann S, Tran M, Robison K, et al. Standardizing hospitalist practice in sepsis and COPD. BMJ Qual Safety. Publication Pending.
5. Colonna S, Sweetenham J, Burgon TB, et al. A better pathway? Building consensus and engaging providers with feedback to improve and standardize cancer care. Clin Breast Cancer. 2018; doi: 10.1016/j.clbc.2018.12.010.
6. Weems L, Strong J, Plummer D, et al. A quality collaboration in heart failure and pneumonia inpatient care at Novant Health: standardizing hospitalist practices to improve patient care and system performance. Jt Comm J Qual Patient Saf. 2018; doi: 10.1016/j.jcjq.2018.09.005.
7. Burgon TB, Cox-Chapman J, Czarnecki C, et al. Engaging Primary Care Providers to Reduce Unwanted Clinical Variation and Support ACO Cost and Quality Goals: A Unique Provider-Payer Collaboration. Popul Health Manag. 2018; doi: 10.1089/pop.2018.0111.
